# Two polymorphic forms of the oxathiin systemic fungicide active carboxine

**DOI:** 10.1107/S2056989018015451

**Published:** 2018-11-09

**Authors:** Christopher S. Frampton, Eleanor S. Frampton, Paul A. Thomson

**Affiliations:** a Experimental Techniques Centre, Brunel University London, Kingston Lane, Uxbridge UB8 3PH, England; b University of Nottingham, University Park, Clifton Blvd, Nottingham NG7 2RD, England; c Vive Crop Protection, 6275 Northam Drive, Unit 1, Mississauga, ON L4V 1Y8, Canada

**Keywords:** crystal structure, polymorphism, fungicide, carboxine, carboxin, hydrogen bonding

## Abstract

Two polymorphic crystal forms of the title compound, C_12_H_13_NO_2_S (systematic name: 6-methyl-*N*-phenyl-2,3-di­hydro-1,4-oxathiine-5-carboxamide), were isolated from a truncated, (12 solvent), polymorph screen on pure lyophillized material. Crystals of form 1 were obtained from all solvents included in the screen with the exception of methanol. As isolated from aceto­nitrile the crystals are triclinic, space group *P*  with *Z′* = 2. Crystals of form 2, which were isolated from methanol only are monoclinic, space group *I*2/*a* with *Z′* = 1. The crystal packing in both structures is dominated by the formation of infinite –NH⋯O hydrogen-bonded chains through the carboxamide core.

## Abstract 2

Two polymorphic crystal forms of 6-methyl-*N*-phenyl-2,3-di­hydro-1,4-oxathiine-5-carboxamide) were isolated from a truncated, (12 solvent), polymorph screen on pure lyophillized material. Crystals of form 1 were obtained from all solvents included in the screen with the exception of methanol. As isolated from aceto­nitrile the crystals are triclinic, space group *P* 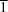 with *Z′* = 2. Crystals of form 2, which were isolated from methanol only are monoclinic, space group *I*2/*a* with *Z′* = 1.

## Chemical context   

6-Methyl-*N*-phenyl-2,3-di­hydro-1,4-oxathiine-5-carboxamide, (Carboxine or Carboxin) **1**, is a systemic fungicide from the oxathiin class of agents. This class of agents was discovered in 1964 (von Schmeling & Kulka, 1966 ▸) and was notable in that they were among the first fungicides that were known to exhibit translocation *i.e.* the ability to move from the leaves to other tissues in the plant. This unique property has made them particularly effective for protection against rusts and smuts. In particular **1**, which is marketed under the trade name VITAVAX^®^, has itself demonstrated high specificity against the fungal class *Basideomycetes*, *Deuteromycetes* and *Phycomycetes* (Edgington *et al.*, 1966 ▸; Edgington & Barron, 1967 ▸; Snel *et al.*, 1970 ▸). There is currently no report of any crystal structure of this important fungicide in the literature although the material has been reported to be dimorphic based upon the observation of two distinct melting points, 91.5–92.5 °C and 98–100 °C (Worthing, 1979 ▸). As part of an ongoing program into the preparation of co-crystal forms of agrichemical active materials to enhance or adapt their physicochemical properties (Eberlin & Frampton, 2017 ▸), it was pertinent to investigate the possible crystal structures of this active material. Given that there is just one hydrogen-bond donor and three possible acceptor groups it was deemed necessary to probe the nature of the hydrogen-bonding inter­actions present in the two distinct forms, thus directing the choice of prospective coformers for a screen. In this paper we report the single crystal X-ray structures of the two reported dimorphic forms of Carboxine **1** at 100 K.
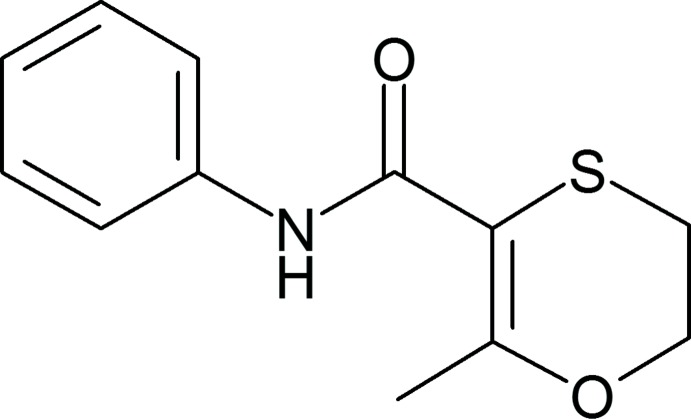


## Structural commentary   

**Figure 1:**
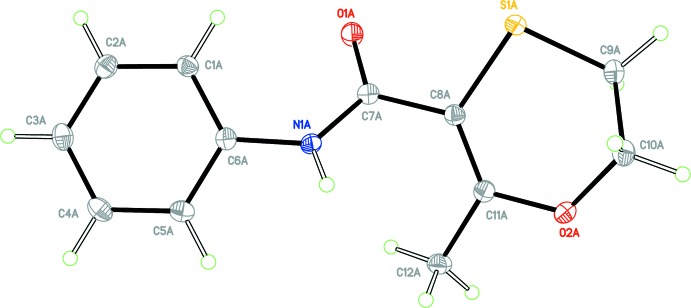
View of mol­ecule *A* of the asymmetric unit of form 1 with the atom labelling scheme. Displacement ellipsoids are drawn at the 50% probability level.

**Figure 3:**
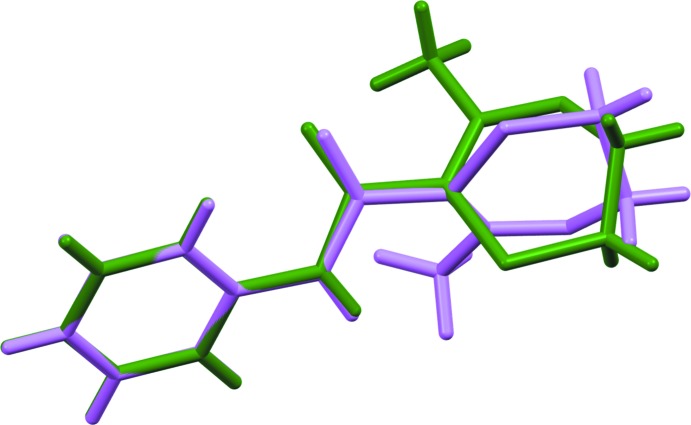
View of the structure overlay of mol­ecule *A* (violet) and mol­ecule *B* (green) from the form 1 structure.

Colourless block-shaped crystals of form 1 were obtained from aceto­nitrile. The crystal structure of form 1 of Carboxine is triclinic, Space group *P* 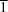, with two independent mol­ecules in the asymmetric unit, (*Z* ’= 2). For clarity, each independent mol­ecule is labelled with suffix *A* or *B*. Figs. 1 ▸ and 2 ▸ show displacement ellipsoid plots for the two mol­ecules, *A* and *B*. Hydrogen-bond distances and angles are given in Table 1 ▸. The mol­ecule contains two rotational degrees of freedom such that the phenyl and oxathiin rings can rotate with respect to the central carboxamide core. The phenyl ring defined by atoms C1–C6 and the carboxamide core defined by atoms C6, N1, C7, O1 and C8 are almost planar. A calculated least-squares plane through the six atoms of the phenyl ring and through the five atoms of the carboxamide core gave r.m.s. deviations from planarity and a calculated dihedral angle between them as follows; Mol­ecule *A*, 0.0016 Å, 0.0278 Å, 24.80 (6)°, respect­ively; mol­ecule *B*, 0.0020 Å, 0.0040 Å, 43.06 (5)°, respectively. It is inter­esting to note that the carboxamide core for Mol­ecule *A* is significantly less planar than that of Mol­ecule *B* with atom N1*A* displaced from the mean plane by −0.0481 (6) Å. The orientation of the oxathiin moiety with respect to the carboxamide core also differs for each mol­ecule in the asymmetric unit with the torsion angle O1—C7—C8—S1 having values of 33.1 (2)° and 143.4 (1)° for mol­ecules *A* and *B*, respectively. Fig. 3 ▸ shows an overlay of the two mol­ecules in the asymmetric unit (Mol­ecule *A* in violet and Mol­ecule *B* in green), showing the differences in their conformations; the overlay was constructed based on the six atoms of the phenyl ring only (r.m.s. deviation = 0.0034 Å) using the Structure Overlay routine in *Mercury* (Macrae *et al.*, 2008 ▸). A DSC of the material from this crystallization experiment gave a single sharp melting endotherm, (onset 97.4 °C, peak 98.2 °C).

**Figure 4:**
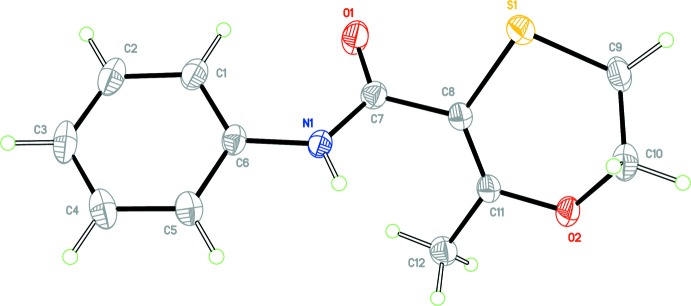
View of mol­ecule 1 of the asymmetric unit of form 2 with the atom labelling scheme. Displacement ellipsoids are drawn at the 50% probability level.

Colourless lath-shaped crystals of form 2 were obtained by slow evaporation from methanol. The crystal structure of form 2 is monoclinic, space group *I*2/*a* with a single mol­ecule in the asymmetric unit, (*Z* ’= 1). Fig. 4 ▸ shows a displacement ellipsoid plot and the hydrogen-bond distance and angle is given in Table 2 ▸. Calculated least-squares planes through the phenyl ring and carboxamide core as described above for the form 1 structure show that these two groups are closer to being coplanar than in the form 1 structure, with r.m.s. deviations from planarity and a calculated dihedral angle between the planes being 0.0064 Å, 0.0154 Å and 9.59 (6)°, respectively. The O1—C7—C8—S1 torsion angle for the form 2 structure is 47.3 (2)°.

**Figure 5:**
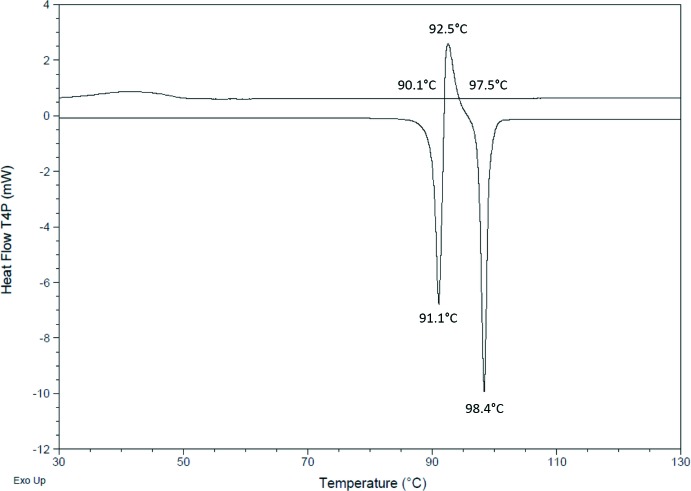
Differential scanning calorimetry thermogram of form 2.

A DSC of the form 2 material is shown in Fig. 5 ▸. It shows a sharp melting endotherm, (onset 90.1 °C, peak 91.1 °C), followed by an exothermic recrystallization event, (onset 92.1 °C, peak 92.5 °C) to form 1, which subsequently gives a sharp melting endotherm (onset 97.5 °C, peak 98.4 °C). From this we deduce that form 1 is the most thermodynamically stable of the two forms, which is also supported by the higher density of form 1 over form 2, 1.431 g cm^−3^ *versus* 1.316 g cm^−3^, respectively. We also note that if crystals of form 2 are left in the methanol mother liquor for a period of time they will spontaneously convert to the form 1 polymorph.

## Supra­molecular features   

**Figure 6:**
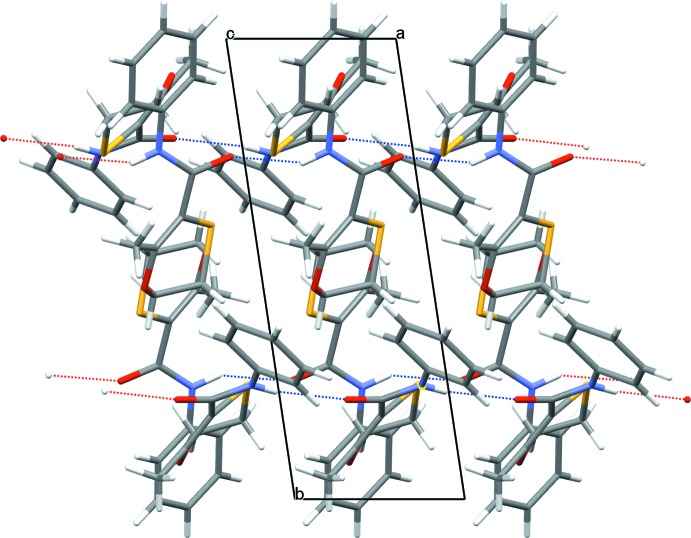
View of the crystal packing of form 1 as viewed approximately down the *a* axis. The N—H⋯O hydrogen bonds are shown as dotted lines (see Table 1 ▸ and text).

**Figure 7:**
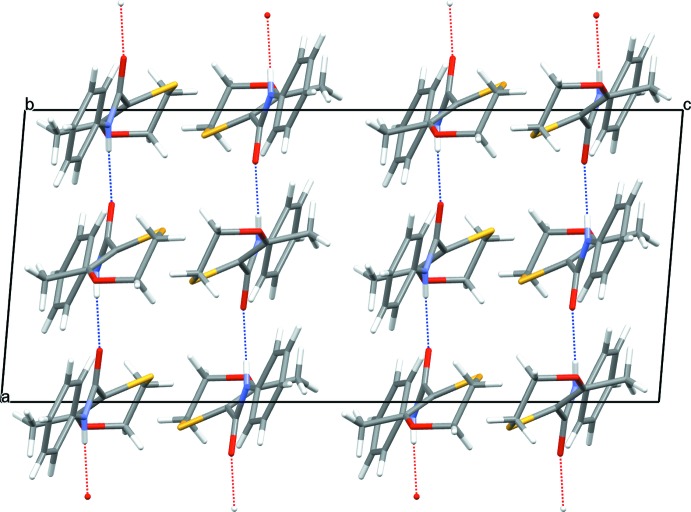
View of the crystal packing of form 2 as viewed down the *b* axis. The N—H⋯O hydrogen bonds are shown as dotted lines (see Table 2 ▸ and text).

The packing of mol­ecules in the crystal structure of form 1 is governed by the formation of two infinite hydrogen-bonded chains, which run parallel to the crystallographic *a* axis, Fig. 6 ▸. These two chains are formed from discrete Mol­ecule *A* and Mol­ecule *B* moieties respectively. The hydrogen-bonding inter­actions are through the amide –NH to carbonyl O for both chains with *D*⋯*A* distances of 2.957 (1) and 2.978 (1) Å for the *A* and *B* chains, respectively. The N—H⋯O hydrogen bond angles for both chains are significantly reduced from 180° to ∼150 (2)° in both chains. The crystal packing found in form 2 is also governed by the formation of an infinite amide –NH to carbonyl O hydrogen-bonded chain, which again runs parallel to the crystallographic *a* axis of the unit cell, Fig. 7 ▸. The *D*⋯*A* distance for this chain is significantly shorter than that found in the form 1 structure at 2.868 (2) Å and the N—H⋯O hydrogen bond angle for this chain is ∼178 (2)°, which is closer to the expected linear value.

## Database survey   

A search of the Cambridge Structural Database (CSD, Version 5.39 update August 2018; Groom *et al.*, 2016 ▸) for the oxathiin moiety yielded just five hits, all of which were genuine examples or analogues of the material under investigation. The closest example to the title compound is the direct dioxide, (–SO_2_), analogue KABFEA (Brown & Baughman, 2010 ▸). A further close example is one where the phenyl group has been substituted at the 4- and 5-positions with a chloro and isopropyl benzoate group, respectively, SOHZUK (Silverton *et al.*, 1991 ▸). Structure ZANDUQ (Kulkarni, 2017 ▸) is a chromene-substituted oxathiin and structure XEQPEO (Caputo *et al.*, 1999 ▸) is an example of a chiral sulfoxide oxathiin with a single phenyl substituent. The remaining example, TUHDUV is a fused oxathiin (Moge *et al.*, 1996 ▸) synthesized in order to incorporate an oxygen atom into tetra­thia­fulvalene.

## Synthesis and crystallization   

Crystals of form 1 and form 2 of Carboxine were isolated from a truncated polymorph screen based on the recrystallization of lyophillized amorphous material from twelve different solvent or solvent water mixtures. Carboxine (Sigma Aldrich, 99.9%, Lot # SZBC023XV), was analyzed by X-ray powder diffraction and DSC as received prior to commencing the polymorph screen. The data demonstrated the starting material to be highly crystalline with a single sharp melting endotherm, (onset 97.4 °C, peak 98.2 °C). This material was assigned as form 1. The polymorph screen consisted of approximately 50 mg of lyophillized Carboxine being dispensed per vial along with approximately 40 volumes of the appropriate solvent or solvent/water mixture (*ca* 2 ml) at room temperature. For the vials that gave clear solutions, these were filtered through a 4 µm filter to remove any potential seeds that may have remained in the solution. Samples that did not dissolve were kept as a slurry. The vials were placed in a platform shaker incubator (Heidolph Titramax/Inkubator 1000) and subjected to a series of heat–cool cycles under shaking from room temperature (RT) to 50 °C (8 h cycles; heating to 50 °C for 4 h and then cooling to RT for a further 4 h) for a maximum of 48 h. The resulting solutions were then allowed to evaporate slowly. Samples that crystallized by saturation crystallization were filtered and the resultant filtrate was then allowed to evaporate to dryness. Samples that did not crystallize were allowed to evaporate to dryness. All solid materials obtained from the screen were analyzed by X-ray powder diffraction. Of the twelve vials in the polymorph screen, eleven demonstrated an X-ray powder diffraction pattern that was identical to that of the starting material (form 1) whereas the material from the twelfth vial gave a pattern that was completely different. Suitable single-crystal samples were selected, form 1 from vial 9, (aceto­nitrile) and form 2 from vial 8 (methanol). A DSC of the form 2 crystalline material was also measured. It should be noted that in the course of this study, it was discovered that if the crystals of form 2 were allowed to remain in the methanol mother liquor, they will over a period of time convert to yield the form 1 structure. A list of solvents and the results of the truncated polymorph screen are given in the supporting information.

## Refinement   

Crystal data, data collection and structure refinement details are summarized in Table 3 ▸. N-bound H atoms were freely refined. C-bound H atoms were positioned geometrically (C—H = 0.95 0.99 Å) and refined as riding with *U*_iso_(H) = 1.2–1.5*U*_eq_(C).

## Supplementary Material

Crystal structure: contains datablock(s) b17006r, b17007r. DOI: 10.1107/S2056989018015451/hb7783sup1.cif

Structure factors: contains datablock(s) b17006r. DOI: 10.1107/S2056989018015451/hb7783b17006rsup2.hkl

Structure factors: contains datablock(s) b17007r. DOI: 10.1107/S2056989018015451/hb7783b17007rsup3.hkl

Supporting information file. DOI: 10.1107/S2056989018015451/hb7783b17006rsup4.cml

Supporting information file. DOI: 10.1107/S2056989018015451/hb7783b17007rsup5.cml

Table of solvents used in the truncated polymorph screen. DOI: 10.1107/S2056989018015451/hb7783sup6.docx

CCDC references: 1876595, 1876594

Additional supporting information:  crystallographic information; 3D view; checkCIF report

## Figures and Tables

**Figure 2:**
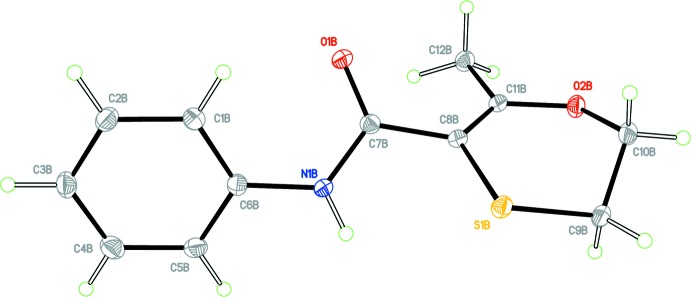
View of mol­ecule *B* of the asymmetric unit of form 1 with the atom labelling scheme. Displacement ellipsoids are drawn at the 50% probability level.

**Table 1:** Hydrogen-bond geometry (Å, °) for form 1

*D*—H⋯*A*	*D*—H	H⋯*A*	*D*⋯*A*	*D*—H⋯*A*
N1*A*—H1*C*⋯O1*A* ^i^	0.860 (18)	2.179 (18)	2.9571 (14)	150.4 (15)
N1*B*—H1*D*⋯O1*B* ^i^	0.84 (2)	2.21 (2)	2.9784 (14)	151.4 (17)

Symmetry code: (i) 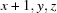.

**Table 2:** Hydrogen-bond geometry (Å, °) for form 2

*D*—H⋯*A*	*D*—H	H⋯*A*	*D*⋯*A*	*D*—H⋯*A*
N1—H1*A*⋯O1^i^	0.87 (2)	2.00 (2)	2.8683 (18)	178.1 (18)

Symmetry code: (i) 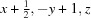.

**Table 3:** Experimental details

	form 1	form 2
Crystal data		
Chemical formula	C_12_H_13_NO_2_S	C_12_H_13_NO_2_S
*M*_r_	235.29	235.29
Crystal system, space group	Triclinic, *P* 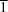	Monoclinic, *I*2/*a*
Temperature (K)	100	100
*a*, *b*, *c* (Å)	5.1669 (2), 14.0781 (5), 15.5152 (5)	9.6424 (2), 11.4059 (3), 21.6672 (5)
α, β, γ (°)	82.596 (3), 80.552 (3), 80.463 (3)	90, 94.711 (2), 90
*V* (Å^3^)	1091.87 (7)	2374.92 (9)
*Z*	4	8
Radiation type	Cu *K*α	Cu *K*α
μ (mm^−1^)	2.51	2.30
Crystal size (mm)	0.56 × 0.20 × 0.14	0.31 × 0.07 × 0.06

Data collection		
Diffractometer	Rigaku SuperNova, Dualflex, AtlasS2	Rigaku SuperNova, Dualflex, AtlasS2
Absorption correction	Analytical (*CrysAlis PRO*; Rigaku OD, 2015 ▸)	Analytical (*CrysAlis PRO*; Rigaku OD, 2015 ▸)
*T*_min_, *T*_max_	0.420, 0.717	0.664, 0.880
No. of measured, independent and observed [*I* > 2σ(*I*)] reflections	8710, 4481, 4393	4644, 2415, 2244
*R*_int_	0.016	0.016
(sin θ/λ)_max_ (Å^−1^)	0.625	0.625

Refinement		
*R*[*F* ^2^ > 2σ(*F* ^2^)], *wR*(*F* ^2^), *S*	0.031, 0.080, 1.03	0.040, 0.116, 1.03
No. of reflections	4481	2415
No. of parameters	299	150
H-atom treatment	H atoms treated by a mixture of independent and constrained refinement	H atoms treated by a mixture of independent and constrained refinement
Δρ_max_, Δρ_min_ (e Å^−3^)	0.32, −0.35	0.51, −0.45

Computer programs: *CrysAlis PRO* (Rigaku OD, 2015 ▸), *SHELXD2014/6* (Schneider & Sheldrick, 2002 ▸), *SHELXL2014/6* (Sheldrick, 2015 ▸), *SHELXTL* (Sheldrick, 2008 ▸) and *Mercury* (Macrae *et al.*, 2008 ▸).

## supplementary crystallographic information

### 6-Methyl-N-phenyl-2,3-dihydro-1,4-oxathiine-5-carboxamide (b17006r) Crystal data

**Table:**

C_12_H_13_NO_2_S	*F*(000) = 496
*M**_r_* = 235.29	*D*_x_ = 1.431 Mg m^−^^3^
Triclinic, *P*1	Melting point: 371.22 K
*a* = 5.1669 (2) Å	Cu *K*α radiation, λ = 1.54178 Å
*b* = 14.0781 (5) Å	Cell parameters from 7281 reflections
*c* = 15.5152 (5) Å	θ = 3.2–76.4°
α = 82.596 (3)°	µ = 2.51 mm^−^^1^
β = 80.552 (3)°	*T* = 100 K
γ = 80.463 (3)°	Block, colourless
*V* = 1091.87 (7) Å^3^	0.56 × 0.20 × 0.14 mm
*Z* = 4	

### 6-Methyl-N-phenyl-2,3-dihydro-1,4-oxathiine-5-carboxamide (b17006r) Data collection

**Table:**

Rigaku SuperNova, Dualflex, AtlasS2 diffractometer	4481 independent reflections
Radiation source: fine-focus sealed X-ray tube, Enhance (Cu) X-ray Source	4393 reflections with *I* > 2σ(*I*)
Detector resolution: 5.2921 pixels mm^-1^	*R*_int_ = 0.016
ω scans	θ_max_ = 74.5°, θ_min_ = 2.9°
Absorption correction: analytical (CrysAlis PRO; Rigaku OD, 2015)	*h* = −5→6
*T*_min_ = 0.420, *T*_max_ = 0.717	*k* = −17→17
8710 measured reflections	*l* = −19→19

### 6-Methyl-N-phenyl-2,3-dihydro-1,4-oxathiine-5-carboxamide (b17006r) Refinement

**Table:**

Refinement on *F*^2^	Primary atom site location: structure-invariant direct methods
Least-squares matrix: full	Hydrogen site location: mixed
*R*[*F*^2^ > 2σ(*F*^2^)] = 0.031	H atoms treated by a mixture of independent and constrained refinement
*wR*(*F*^2^) = 0.080	*w* = 1/[σ^2^(*F*_o_^2^) + (0.0425*P*)^2^ + 0.650*P*] where *P* = (*F*_o_^2^ + 2*F*_c_^2^)/3
*S* = 1.03	(Δ/σ)_max_ = 0.001
4481 reflections	Δρ_max_ = 0.32 e Å^−^^3^
299 parameters	Δρ_min_ = −0.35 e Å^−^^3^
0 restraints	

### 6-Methyl-N-phenyl-2,3-dihydro-1,4-oxathiine-5-carboxamide (b17006r) Special details

**Table:**

Geometry. All esds (except the esd in the dihedral angle between two l.s. planes) are estimated using the full covariance matrix. The cell esds are taken into account individually in the estimation of esds in distances, angles and torsion angles; correlations between esds in cell parameters are only used when they are defined by crystal symmetry. An approximate (isotropic) treatment of cell esds is used for estimating esds involving l.s. planes.

### 6-Methyl-N-phenyl-2,3-dihydro-1,4-oxathiine-5-carboxamide (b17006r) Fractional atomic coordinates and isotropic or equivalent isotropic displacement parameters (Å^2^)

**Table:**

	*x*	*y*	*z*	*U*_iso_*/*U*_eq_	
S1A	0.24969 (6)	0.59541 (2)	0.62071 (2)	0.01479 (9)	
O1A	0.07910 (18)	0.74358 (7)	0.74003 (7)	0.0169 (2)	
O2A	0.70130 (18)	0.45917 (7)	0.71133 (6)	0.01512 (19)	
N1A	0.4950 (2)	0.75662 (8)	0.76391 (7)	0.0127 (2)	
H1C	0.659 (4)	0.7308 (12)	0.7563 (11)	0.013 (4)*	
C1A	0.2283 (3)	0.91659 (9)	0.78592 (8)	0.0135 (2)	
H1A	0.1107	0.9068	0.7479	0.016*	
C2A	0.1908 (3)	1.00263 (10)	0.82504 (9)	0.0155 (3)	
H2A	0.0465	1.0514	0.8135	0.019*	
C3A	0.3614 (3)	1.01785 (10)	0.88044 (9)	0.0163 (3)	
H3A	0.3348	1.0768	0.9065	0.020*	
C4A	0.5717 (3)	0.94615 (10)	0.89754 (9)	0.0161 (3)	
H4A	0.6890	0.9562	0.9356	0.019*	
C5A	0.6115 (3)	0.85993 (10)	0.85930 (8)	0.0140 (3)	
H5A	0.7552	0.8111	0.8713	0.017*	
C6A	0.4398 (2)	0.84526 (9)	0.80313 (8)	0.0121 (2)	
C7A	0.3145 (3)	0.70923 (9)	0.73973 (8)	0.0124 (2)	
C8A	0.4183 (2)	0.61317 (9)	0.70683 (8)	0.0123 (2)	
C9A	0.3579 (3)	0.46729 (9)	0.61878 (9)	0.0159 (3)	
H9A	0.2544	0.4315	0.6680	0.019*	
H9B	0.3263	0.4469	0.5631	0.019*	
C10A	0.6506 (3)	0.44334 (10)	0.62671 (9)	0.0176 (3)	
H10A	0.7133	0.3747	0.6168	0.021*	
H10B	0.7515	0.4841	0.5808	0.021*	
C11A	0.6002 (2)	0.54610 (9)	0.74270 (8)	0.0127 (2)	
C12A	0.7129 (3)	0.54989 (10)	0.82493 (9)	0.0165 (3)	
H12A	0.7204	0.4864	0.8595	0.025*	
H12B	0.5999	0.5987	0.8596	0.025*	
H12C	0.8922	0.5671	0.8096	0.025*	
S1B	0.82208 (6)	0.75132 (2)	0.43964 (2)	0.01312 (9)	
O1B	0.39731 (18)	0.78370 (7)	0.25355 (6)	0.0160 (2)	
O2B	0.35235 (18)	0.91997 (7)	0.48272 (6)	0.01362 (19)	
N1B	0.8469 (2)	0.75216 (8)	0.24445 (7)	0.0120 (2)	
H1D	0.975 (4)	0.7656 (13)	0.2653 (12)	0.022 (5)*	
C1B	0.7445 (3)	0.65546 (9)	0.13678 (9)	0.0141 (3)	
H1B	0.5999	0.6354	0.1773	0.017*	
C2B	0.8057 (3)	0.62198 (10)	0.05419 (9)	0.0168 (3)	
H2B	0.7029	0.5785	0.0387	0.020*	
C3B	1.0153 (3)	0.65141 (11)	−0.00591 (9)	0.0180 (3)	
H3B	1.0555	0.6284	−0.0622	0.022*	
C4B	1.1653 (3)	0.71480 (11)	0.01725 (9)	0.0185 (3)	
H4B	1.3085	0.7354	−0.0236	0.022*	
C5B	1.1079 (3)	0.74833 (10)	0.09968 (9)	0.0152 (3)	
H5B	1.2121	0.7913	0.1152	0.018*	
C6B	0.8970 (2)	0.71870 (9)	0.15961 (8)	0.0122 (2)	
C7B	0.5989 (3)	0.78364 (9)	0.28597 (8)	0.0119 (2)	
C8B	0.5859 (2)	0.81617 (9)	0.37465 (8)	0.0116 (2)	
C9B	0.7615 (3)	0.82802 (10)	0.52748 (8)	0.0143 (3)	
H9C	0.8372	0.7928	0.5792	0.017*	
H9D	0.8485	0.8864	0.5086	0.017*	
C10B	0.4647 (3)	0.85779 (10)	0.55196 (8)	0.0145 (3)	
H10C	0.3769	0.7991	0.5652	0.017*	
H10D	0.4313	0.8917	0.6056	0.017*	
C11B	0.3986 (2)	0.88970 (9)	0.40068 (8)	0.0118 (2)	
C12B	0.2180 (3)	0.95418 (10)	0.34453 (9)	0.0143 (3)	
H12D	0.0459	0.9310	0.3534	0.021*	
H12E	0.2965	0.9534	0.2826	0.021*	
H12F	0.1932	1.0205	0.3607	0.021*	

### 6-Methyl-N-phenyl-2,3-dihydro-1,4-oxathiine-5-carboxamide (b17006r) Atomic displacement parameters (Å^2^)

**Table:**

	*U*^11^	*U*^22^	*U*^33^	*U*^12^	*U*^13^	*U*^23^
S1A	0.01540 (16)	0.01280 (15)	0.01776 (16)	−0.00099 (12)	−0.00729 (12)	−0.00270 (11)
O1A	0.0094 (4)	0.0170 (5)	0.0254 (5)	−0.0008 (4)	−0.0028 (4)	−0.0070 (4)
O2A	0.0142 (4)	0.0125 (4)	0.0186 (5)	0.0010 (3)	−0.0046 (4)	−0.0025 (4)
N1A	0.0086 (5)	0.0125 (5)	0.0170 (5)	0.0003 (4)	−0.0024 (4)	−0.0035 (4)
C1A	0.0126 (6)	0.0142 (6)	0.0140 (6)	−0.0028 (5)	−0.0025 (5)	−0.0012 (5)
C2A	0.0145 (6)	0.0144 (6)	0.0166 (6)	−0.0011 (5)	−0.0009 (5)	−0.0013 (5)
C3A	0.0190 (7)	0.0157 (6)	0.0146 (6)	−0.0041 (5)	0.0006 (5)	−0.0047 (5)
C4A	0.0161 (6)	0.0214 (7)	0.0121 (6)	−0.0057 (5)	−0.0021 (5)	−0.0033 (5)
C5A	0.0114 (6)	0.0176 (6)	0.0126 (6)	−0.0020 (5)	−0.0009 (5)	−0.0010 (5)
C6A	0.0118 (6)	0.0125 (6)	0.0120 (6)	−0.0033 (5)	0.0002 (5)	−0.0010 (5)
C7A	0.0116 (6)	0.0134 (6)	0.0120 (6)	−0.0022 (5)	−0.0010 (5)	−0.0007 (5)
C8A	0.0102 (6)	0.0133 (6)	0.0138 (6)	−0.0027 (5)	−0.0015 (5)	−0.0020 (5)
C9A	0.0181 (7)	0.0128 (6)	0.0179 (6)	−0.0022 (5)	−0.0037 (5)	−0.0041 (5)
C10A	0.0176 (7)	0.0181 (6)	0.0171 (6)	−0.0002 (5)	−0.0016 (5)	−0.0060 (5)
C11A	0.0104 (6)	0.0126 (6)	0.0150 (6)	−0.0026 (5)	−0.0006 (5)	−0.0016 (5)
C12A	0.0168 (6)	0.0167 (6)	0.0166 (6)	−0.0017 (5)	−0.0065 (5)	0.0003 (5)
S1B	0.01241 (15)	0.01383 (15)	0.01324 (15)	0.00139 (11)	−0.00400 (11)	−0.00350 (11)
O1B	0.0107 (4)	0.0232 (5)	0.0160 (4)	−0.0026 (4)	−0.0030 (4)	−0.0075 (4)
O2B	0.0139 (4)	0.0158 (4)	0.0110 (4)	0.0011 (4)	−0.0027 (3)	−0.0039 (3)
N1B	0.0095 (5)	0.0157 (5)	0.0119 (5)	−0.0017 (4)	−0.0026 (4)	−0.0043 (4)
C1B	0.0128 (6)	0.0150 (6)	0.0145 (6)	−0.0012 (5)	−0.0014 (5)	−0.0030 (5)
C2B	0.0157 (6)	0.0181 (6)	0.0180 (6)	−0.0006 (5)	−0.0057 (5)	−0.0059 (5)
C3B	0.0173 (7)	0.0236 (7)	0.0125 (6)	0.0023 (5)	−0.0033 (5)	−0.0056 (5)
C4B	0.0144 (6)	0.0255 (7)	0.0144 (6)	−0.0024 (5)	−0.0002 (5)	−0.0006 (5)
C5B	0.0122 (6)	0.0181 (6)	0.0159 (6)	−0.0025 (5)	−0.0037 (5)	−0.0014 (5)
C6B	0.0117 (6)	0.0123 (6)	0.0121 (6)	0.0016 (5)	−0.0032 (5)	−0.0019 (5)
C7B	0.0118 (6)	0.0111 (6)	0.0130 (6)	−0.0025 (4)	−0.0014 (5)	−0.0016 (4)
C8B	0.0102 (6)	0.0138 (6)	0.0115 (6)	−0.0032 (5)	−0.0021 (4)	−0.0017 (5)
C9B	0.0140 (6)	0.0183 (6)	0.0115 (6)	−0.0014 (5)	−0.0037 (5)	−0.0043 (5)
C10B	0.0146 (6)	0.0185 (6)	0.0102 (6)	−0.0012 (5)	−0.0029 (5)	−0.0013 (5)
C11B	0.0109 (6)	0.0141 (6)	0.0114 (6)	−0.0041 (5)	−0.0020 (5)	−0.0023 (5)
C12B	0.0137 (6)	0.0155 (6)	0.0137 (6)	−0.0001 (5)	−0.0036 (5)	−0.0023 (5)

### 6-Methyl-N-phenyl-2,3-dihydro-1,4-oxathiine-5-carboxamide (b17006r) Geometric parameters (Å, º)

**Table:**

S1A—C8A	1.7741 (13)	S1B—C8B	1.7712 (13)
S1A—C9A	1.8003 (13)	S1B—C9B	1.8024 (13)
O1A—C7A	1.2317 (16)	O1B—C7B	1.2287 (16)
O2A—C11A	1.3658 (16)	O2B—C11B	1.3657 (15)
O2A—C10A	1.4316 (16)	O2B—C10B	1.4323 (15)
N1A—C7A	1.3571 (17)	N1B—C7B	1.3666 (17)
N1A—C6A	1.4247 (16)	N1B—C6B	1.4244 (16)
N1A—H1C	0.860 (18)	N1B—H1D	0.84 (2)
C1A—C6A	1.3917 (18)	C1B—C2B	1.3919 (18)
C1A—C2A	1.3951 (18)	C1B—C6B	1.3945 (18)
C1A—H1A	0.9500	C1B—H1B	0.9500
C2A—C3A	1.3858 (19)	C2B—C3B	1.390 (2)
C2A—H2A	0.9500	C2B—H2B	0.9500
C3A—C4A	1.390 (2)	C3B—C4B	1.389 (2)
C3A—H3A	0.9500	C3B—H3B	0.9500
C4A—C5A	1.3895 (19)	C4B—C5B	1.3896 (19)
C4A—H4A	0.9500	C4B—H4B	0.9500
C5A—C6A	1.3965 (18)	C5B—C6B	1.3943 (18)
C5A—H5A	0.9500	C5B—H5B	0.9500
C7A—C8A	1.4915 (17)	C7B—C8B	1.4930 (17)
C8A—C11A	1.3517 (18)	C8B—C11B	1.3486 (18)
C9A—C10A	1.5159 (19)	C9B—C10B	1.5190 (18)
C9A—H9A	0.9900	C9B—H9C	0.9900
C9A—H9B	0.9900	C9B—H9D	0.9900
C10A—H10A	0.9900	C10B—H10C	0.9900
C10A—H10B	0.9900	C10B—H10D	0.9900
C11A—C12A	1.4971 (18)	C11B—C12B	1.4953 (17)
C12A—H12A	0.9800	C12B—H12D	0.9800
C12A—H12B	0.9800	C12B—H12E	0.9800
C12A—H12C	0.9800	C12B—H12F	0.9800
			
C8A—S1A—C9A	97.81 (6)	C8B—S1B—C9B	98.52 (6)
C11A—O2A—C10A	118.67 (10)	C11B—O2B—C10B	118.26 (10)
C7A—N1A—C6A	126.29 (11)	C7B—N1B—C6B	123.68 (11)
C7A—N1A—H1C	117.9 (11)	C7B—N1B—H1D	116.5 (13)
C6A—N1A—H1C	115.8 (11)	C6B—N1B—H1D	118.2 (13)
C6A—C1A—C2A	119.32 (12)	C2B—C1B—C6B	119.48 (12)
C6A—C1A—H1A	120.3	C2B—C1B—H1B	120.3
C2A—C1A—H1A	120.3	C6B—C1B—H1B	120.3
C3A—C2A—C1A	120.87 (13)	C3B—C2B—C1B	120.82 (13)
C3A—C2A—H2A	119.6	C3B—C2B—H2B	119.6
C1A—C2A—H2A	119.6	C1B—C2B—H2B	119.6
C2A—C3A—C4A	119.48 (12)	C4B—C3B—C2B	119.30 (12)
C2A—C3A—H3A	120.3	C4B—C3B—H3B	120.4
C4A—C3A—H3A	120.3	C2B—C3B—H3B	120.4
C5A—C4A—C3A	120.38 (12)	C3B—C4B—C5B	120.57 (13)
C5A—C4A—H4A	119.8	C3B—C4B—H4B	119.7
C3A—C4A—H4A	119.8	C5B—C4B—H4B	119.7
C4A—C5A—C6A	119.86 (12)	C4B—C5B—C6B	119.85 (12)
C4A—C5A—H5A	120.1	C4B—C5B—H5B	120.1
C6A—C5A—H5A	120.1	C6B—C5B—H5B	120.1
C1A—C6A—C5A	120.08 (12)	C5B—C6B—C1B	119.98 (12)
C1A—C6A—N1A	122.60 (12)	C5B—C6B—N1B	118.67 (12)
C5A—C6A—N1A	117.30 (12)	C1B—C6B—N1B	121.32 (12)
O1A—C7A—N1A	123.41 (12)	O1B—C7B—N1B	122.47 (12)
O1A—C7A—C8A	120.18 (12)	O1B—C7B—C8B	121.54 (11)
N1A—C7A—C8A	116.32 (11)	N1B—C7B—C8B	115.97 (11)
C11A—C8A—C7A	124.36 (12)	C11B—C8B—C7B	119.59 (11)
C11A—C8A—S1A	124.84 (10)	C11B—C8B—S1B	124.58 (10)
C7A—C8A—S1A	110.59 (9)	C7B—C8B—S1B	115.82 (9)
C10A—C9A—S1A	110.23 (9)	C10B—C9B—S1B	109.60 (9)
C10A—C9A—H9A	109.6	C10B—C9B—H9C	109.8
S1A—C9A—H9A	109.6	S1B—C9B—H9C	109.8
C10A—C9A—H9B	109.6	C10B—C9B—H9D	109.8
S1A—C9A—H9B	109.6	S1B—C9B—H9D	109.8
H9A—C9A—H9B	108.1	H9C—C9B—H9D	108.2
O2A—C10A—C9A	111.67 (11)	O2B—C10B—C9B	111.73 (10)
O2A—C10A—H10A	109.3	O2B—C10B—H10C	109.3
C9A—C10A—H10A	109.3	C9B—C10B—H10C	109.3
O2A—C10A—H10B	109.3	O2B—C10B—H10D	109.3
C9A—C10A—H10B	109.3	C9B—C10B—H10D	109.3
H10A—C10A—H10B	107.9	H10C—C10B—H10D	107.9
C8A—C11A—O2A	124.57 (12)	C8B—C11B—O2B	124.79 (12)
C8A—C11A—C12A	127.17 (12)	C8B—C11B—C12B	126.26 (12)
O2A—C11A—C12A	108.16 (11)	O2B—C11B—C12B	108.88 (11)
C11A—C12A—H12A	109.5	C11B—C12B—H12D	109.5
C11A—C12A—H12B	109.5	C11B—C12B—H12E	109.5
H12A—C12A—H12B	109.5	H12D—C12B—H12E	109.5
C11A—C12A—H12C	109.5	C11B—C12B—H12F	109.5
H12A—C12A—H12C	109.5	H12D—C12B—H12F	109.5
H12B—C12A—H12C	109.5	H12E—C12B—H12F	109.5
			
C6A—C1A—C2A—C3A	0.1 (2)	C6B—C1B—C2B—C3B	−0.5 (2)
C1A—C2A—C3A—C4A	−0.3 (2)	C1B—C2B—C3B—C4B	0.2 (2)
C2A—C3A—C4A—C5A	0.2 (2)	C2B—C3B—C4B—C5B	0.2 (2)
C3A—C4A—C5A—C6A	0.2 (2)	C3B—C4B—C5B—C6B	−0.4 (2)
C2A—C1A—C6A—C5A	0.25 (19)	C4B—C5B—C6B—C1B	0.1 (2)
C2A—C1A—C6A—N1A	−178.34 (12)	C4B—C5B—C6B—N1B	178.37 (12)
C4A—C5A—C6A—C1A	−0.42 (19)	C2B—C1B—C6B—C5B	0.37 (19)
C4A—C5A—C6A—N1A	178.24 (11)	C2B—C1B—C6B—N1B	−177.87 (12)
C7A—N1A—C6A—C1A	−30.94 (19)	C7B—N1B—C6B—C5B	137.13 (13)
C7A—N1A—C6A—C5A	150.44 (13)	C7B—N1B—C6B—C1B	−44.61 (18)
C6A—N1A—C7A—O1A	8.5 (2)	C6B—N1B—C7B—O1B	1.5 (2)
C6A—N1A—C7A—C8A	−175.00 (11)	C6B—N1B—C7B—C8B	−179.89 (11)
O1A—C7A—C8A—C11A	−141.95 (14)	O1B—C7B—C8B—C11B	−35.57 (18)
N1A—C7A—C8A—C11A	41.41 (18)	N1B—C7B—C8B—C11B	145.77 (12)
O1A—C7A—C8A—S1A	33.06 (15)	O1B—C7B—C8B—S1B	143.35 (11)
N1A—C7A—C8A—S1A	−143.58 (10)	N1B—C7B—C8B—S1B	−35.30 (14)
C9A—S1A—C8A—C11A	9.14 (13)	C9B—S1B—C8B—C11B	−9.94 (13)
C9A—S1A—C8A—C7A	−165.85 (9)	C9B—S1B—C8B—C7B	171.20 (9)
C8A—S1A—C9A—C10A	−42.83 (11)	C8B—S1B—C9B—C10B	42.24 (10)
C11A—O2A—C10A—C9A	−50.88 (15)	C11B—O2B—C10B—C9B	53.01 (15)
S1A—C9A—C10A—O2A	67.27 (13)	S1B—C9B—C10B—O2B	−67.15 (12)
C7A—C8A—C11A—O2A	−178.09 (11)	C7B—C8B—C11B—O2B	173.91 (11)
S1A—C8A—C11A—O2A	7.60 (19)	S1B—C8B—C11B—O2B	−4.91 (19)
C7A—C8A—C11A—C12A	6.0 (2)	C7B—C8B—C11B—C12B	−9.3 (2)
S1A—C8A—C11A—C12A	−168.29 (10)	S1B—C8B—C11B—C12B	171.87 (10)
C10A—O2A—C11A—C8A	11.62 (18)	C10B—O2B—C11B—C8B	−14.86 (18)
C10A—O2A—C11A—C12A	−171.82 (11)	C10B—O2B—C11B—C12B	167.87 (10)

### 6-Methyl-N-phenyl-2,3-dihydro-1,4-oxathiine-5-carboxamide (b17006r) Hydrogen-bond geometry (Å, º)

**Table:**

*D*—H···*A*	*D*—H	H···*A*	*D*···*A*	*D*—H···*A*
N1*A*—H1*C*···O1*A*^i^	0.860 (18)	2.179 (18)	2.9571 (14)	150.4 (15)
N1*B*—H1*D*···O1*B*^i^	0.84 (2)	2.21 (2)	2.9784 (14)	151.4 (17)

Symmetry code: (i) *x*+1, *y*, *z*.

### 6-methyl-N-phenyl-2,3-dihydro-1,4-oxathiine-5-carboxamide (b17007r) Crystal data

**Table:**

C_12_H_13_NO_2_S	*D*_x_ = 1.316 Mg m^−^^3^
*M**_r_* = 235.29	Melting point: 364.13 K
Monoclinic, *I*2/*a*	Cu *K*α radiation, λ = 1.54178 Å
*a* = 9.6424 (2) Å	Cell parameters from 2580 reflections
*b* = 11.4059 (3) Å	θ = 4.1–75.8°
*c* = 21.6672 (5) Å	µ = 2.30 mm^−^^1^
β = 94.711 (2)°	*T* = 100 K
*V* = 2374.92 (9) Å^3^	Plate, colourless
*Z* = 8	0.31 × 0.07 × 0.06 mm
*F*(000) = 992	

### 6-methyl-N-phenyl-2,3-dihydro-1,4-oxathiine-5-carboxamide (b17007r) Data collection

**Table:**

Rigaku SuperNova, Dualflex, AtlasS2 diffractometer	2415 independent reflections
Radiation source: fine-focus sealed X-ray tube, Enhance (Cu) X-ray Source	2244 reflections with *I* > 2σ(*I*)
Detector resolution: 5.2921 pixels mm^-1^	*R*_int_ = 0.016
ω scans	θ_max_ = 74.5°, θ_min_ = 4.1°
Absorption correction: analytical (CrysAlis PRO; Rigaku OD, 2015)	*h* = −8→11
*T*_min_ = 0.664, *T*_max_ = 0.880	*k* = −14→12
4644 measured reflections	*l* = −26→27

### 6-methyl-N-phenyl-2,3-dihydro-1,4-oxathiine-5-carboxamide (b17007r) Refinement

**Table:**

Refinement on *F*^2^	0 restraints
Least-squares matrix: full	Hydrogen site location: mixed
*R*[*F*^2^ > 2σ(*F*^2^)] = 0.040	H atoms treated by a mixture of independent and constrained refinement
*wR*(*F*^2^) = 0.116	*w* = 1/[σ^2^(*F*_o_^2^) + (0.070*P*)^2^ + 2.2*P*] where *P* = (*F*_o_^2^ + 2*F*_c_^2^)/3
*S* = 1.03	(Δ/σ)_max_ < 0.001
2415 reflections	Δρ_max_ = 0.51 e Å^−^^3^
150 parameters	Δρ_min_ = −0.44 e Å^−^^3^

### 6-methyl-N-phenyl-2,3-dihydro-1,4-oxathiine-5-carboxamide (b17007r) Special details

**Table:**

Geometry. All esds (except the esd in the dihedral angle between two l.s. planes) are estimated using the full covariance matrix. The cell esds are taken into account individually in the estimation of esds in distances, angles and torsion angles; correlations between esds in cell parameters are only used when they are defined by crystal symmetry. An approximate (isotropic) treatment of cell esds is used for estimating esds involving l.s. planes.

### 6-methyl-N-phenyl-2,3-dihydro-1,4-oxathiine-5-carboxamide (b17007r) Fractional atomic coordinates and isotropic or equivalent isotropic displacement parameters (Å^2^)

**Table:**

	*x*	*y*	*z*	*U*_iso_*/*U*_eq_	
S1	0.41282 (5)	0.67311 (4)	0.72445 (2)	0.03174 (16)	
O1	0.32397 (12)	0.46505 (10)	0.64353 (6)	0.0322 (3)	
O2	0.58438 (12)	0.80835 (10)	0.62859 (5)	0.0252 (3)	
N1	0.55181 (13)	0.43217 (11)	0.62844 (6)	0.0208 (3)	
H1A	0.635 (2)	0.4633 (18)	0.6321 (9)	0.023 (5)*	
C1	0.43269 (18)	0.23962 (14)	0.61678 (8)	0.0269 (3)	
H1	0.3512	0.2694	0.6330	0.032*	
C2	0.4391 (2)	0.12309 (15)	0.59833 (9)	0.0333 (4)	
H2	0.3604	0.0739	0.6012	0.040*	
C3	0.5581 (2)	0.07730 (15)	0.57582 (8)	0.0338 (4)	
H3	0.5612	−0.0025	0.5635	0.041*	
C4	0.6725 (2)	0.14945 (16)	0.57147 (8)	0.0318 (4)	
H4	0.7551	0.1185	0.5568	0.038*	
C5	0.66723 (17)	0.26668 (15)	0.58839 (8)	0.0260 (3)	
H5	0.7455	0.3159	0.5845	0.031*	
C6	0.54737 (16)	0.31217 (13)	0.61114 (7)	0.0213 (3)	
C7	0.44398 (16)	0.50099 (14)	0.64111 (7)	0.0218 (3)	
C8	0.48110 (15)	0.62578 (13)	0.65536 (7)	0.0207 (3)	
C9	0.4412 (2)	0.82819 (14)	0.71537 (9)	0.0315 (4)	
H9A	0.3670	0.8613	0.6862	0.038*	
H9B	0.4372	0.8680	0.7558	0.038*	
C10	0.58140 (19)	0.84960 (15)	0.69104 (8)	0.0297 (4)	
H10A	0.6022	0.9346	0.6925	0.036*	
H10B	0.6543	0.8089	0.7179	0.036*	
C11	0.54640 (16)	0.69442 (13)	0.61652 (7)	0.0206 (3)	
C12	0.57867 (18)	0.66346 (14)	0.55218 (8)	0.0262 (3)	
H12A	0.5424	0.7246	0.5234	0.039*	
H12B	0.5351	0.5883	0.5403	0.039*	
H12C	0.6797	0.6572	0.5506	0.039*	

### 6-methyl-N-phenyl-2,3-dihydro-1,4-oxathiine-5-carboxamide (b17007r) Atomic displacement parameters (Å^2^)

**Table:**

	*U*^11^	*U*^22^	*U*^33^	*U*^12^	*U*^13^	*U*^23^
S1	0.0473 (3)	0.0234 (2)	0.0265 (2)	−0.00202 (16)	0.01526 (18)	−0.00236 (14)
O1	0.0212 (6)	0.0206 (6)	0.0553 (8)	−0.0003 (4)	0.0062 (5)	−0.0025 (5)
O2	0.0341 (6)	0.0158 (5)	0.0265 (6)	−0.0016 (4)	0.0064 (5)	−0.0005 (4)
N1	0.0207 (6)	0.0159 (6)	0.0258 (7)	−0.0012 (5)	0.0031 (5)	−0.0007 (5)
C1	0.0309 (8)	0.0199 (8)	0.0298 (8)	−0.0033 (6)	0.0027 (6)	0.0014 (6)
C2	0.0454 (10)	0.0196 (8)	0.0342 (9)	−0.0061 (7)	−0.0011 (7)	0.0012 (7)
C3	0.0531 (11)	0.0164 (7)	0.0306 (9)	0.0043 (7)	−0.0045 (7)	−0.0010 (6)
C4	0.0414 (9)	0.0243 (8)	0.0290 (8)	0.0106 (7)	−0.0009 (7)	−0.0035 (7)
C5	0.0279 (8)	0.0224 (8)	0.0272 (8)	0.0033 (6)	−0.0005 (6)	−0.0011 (6)
C6	0.0267 (8)	0.0160 (7)	0.0207 (7)	0.0013 (6)	−0.0004 (6)	0.0011 (5)
C7	0.0228 (7)	0.0191 (7)	0.0235 (7)	0.0002 (5)	0.0019 (6)	0.0011 (6)
C8	0.0225 (7)	0.0177 (7)	0.0220 (7)	0.0013 (5)	0.0026 (5)	−0.0016 (5)
C9	0.0484 (10)	0.0199 (8)	0.0272 (9)	0.0019 (7)	0.0089 (7)	−0.0062 (6)
C10	0.0407 (9)	0.0208 (7)	0.0269 (8)	−0.0021 (7)	−0.0011 (7)	−0.0040 (6)
C11	0.0223 (7)	0.0167 (7)	0.0230 (7)	0.0023 (5)	0.0023 (5)	−0.0008 (6)
C12	0.0337 (8)	0.0232 (8)	0.0222 (8)	0.0023 (6)	0.0055 (6)	0.0011 (6)

### 6-methyl-N-phenyl-2,3-dihydro-1,4-oxathiine-5-carboxamide (b17007r) Geometric parameters (Å, º)

**Table:**

S1—C8	1.7684 (15)	C4—C5	1.388 (2)
S1—C9	1.8031 (17)	C4—H4	0.9500
O1—C7	1.2328 (19)	C5—C6	1.393 (2)
O2—C11	1.3694 (19)	C5—H5	0.9500
O2—C10	1.435 (2)	C7—C8	1.494 (2)
N1—C7	1.349 (2)	C8—C11	1.343 (2)
N1—C6	1.4188 (19)	C9—C10	1.511 (3)
N1—H1A	0.87 (2)	C9—H9A	0.9900
C1—C2	1.391 (2)	C9—H9B	0.9900
C1—C6	1.395 (2)	C10—H10A	0.9900
C1—H1	0.9500	C10—H10B	0.9900
C2—C3	1.385 (3)	C11—C12	1.496 (2)
C2—H2	0.9500	C12—H12A	0.9800
C3—C4	1.385 (3)	C12—H12B	0.9800
C3—H3	0.9500	C12—H12C	0.9800
			
C8—S1—C9	97.85 (8)	N1—C7—C8	114.90 (13)
C11—O2—C10	117.73 (13)	C11—C8—C7	122.85 (14)
C7—N1—C6	127.45 (13)	C11—C8—S1	125.35 (12)
C7—N1—H1A	117.4 (13)	C7—C8—S1	111.49 (11)
C6—N1—H1A	115.1 (13)	C10—C9—S1	110.17 (12)
C2—C1—C6	119.17 (16)	C10—C9—H9A	109.6
C2—C1—H1	120.4	S1—C9—H9A	109.6
C6—C1—H1	120.4	C10—C9—H9B	109.6
C3—C2—C1	121.30 (17)	S1—C9—H9B	109.6
C3—C2—H2	119.3	H9A—C9—H9B	108.1
C1—C2—H2	119.3	O2—C10—C9	111.33 (14)
C2—C3—C4	119.13 (16)	O2—C10—H10A	109.4
C2—C3—H3	120.4	C9—C10—H10A	109.4
C4—C3—H3	120.4	O2—C10—H10B	109.4
C3—C4—C5	120.52 (17)	C9—C10—H10B	109.4
C3—C4—H4	119.7	H10A—C10—H10B	108.0
C5—C4—H4	119.7	C8—C11—O2	124.41 (14)
C4—C5—C6	120.08 (16)	C8—C11—C12	126.19 (14)
C4—C5—H5	120.0	O2—C11—C12	109.22 (13)
C6—C5—H5	120.0	C11—C12—H12A	109.5
C5—C6—C1	119.78 (15)	C11—C12—H12B	109.5
C5—C6—N1	116.41 (14)	H12A—C12—H12B	109.5
C1—C6—N1	123.80 (14)	C11—C12—H12C	109.5
O1—C7—N1	123.86 (15)	H12A—C12—H12C	109.5
O1—C7—C8	121.19 (14)	H12B—C12—H12C	109.5
			
C6—C1—C2—C3	1.4 (3)	O1—C7—C8—S1	47.26 (19)
C1—C2—C3—C4	−0.2 (3)	N1—C7—C8—S1	−130.00 (12)
C2—C3—C4—C5	−1.2 (3)	C9—S1—C8—C11	6.79 (16)
C3—C4—C5—C6	1.3 (3)	C9—S1—C8—C7	−166.98 (12)
C4—C5—C6—C1	−0.1 (2)	C8—S1—C9—C10	−41.24 (14)
C4—C5—C6—N1	178.84 (14)	C11—O2—C10—C9	−53.62 (19)
C2—C1—C6—C5	−1.3 (2)	S1—C9—C10—O2	67.88 (16)
C2—C1—C6—N1	179.90 (15)	C7—C8—C11—O2	−178.68 (14)
C7—N1—C6—C5	167.39 (15)	S1—C8—C11—O2	8.2 (2)
C7—N1—C6—C1	−13.7 (3)	C7—C8—C11—C12	6.6 (2)
C6—N1—C7—O1	5.4 (3)	S1—C8—C11—C12	−166.46 (12)
C6—N1—C7—C8	−177.45 (14)	C10—O2—C11—C8	13.7 (2)
O1—C7—C8—C11	−126.69 (18)	C10—O2—C11—C12	−170.79 (13)
N1—C7—C8—C11	56.1 (2)		

### 6-methyl-N-phenyl-2,3-dihydro-1,4-oxathiine-5-carboxamide (b17007r) Hydrogen-bond geometry (Å, º)

**Table:**

*D*—H···*A*	*D*—H	H···*A*	*D*···*A*	*D*—H···*A*
N1—H1*A*···O1^i^	0.87 (2)	2.00 (2)	2.8683 (18)	178.1 (18)

Symmetry code: (i) *x*+1/2, −*y*+1, *z*.
