# Supplementary material for: Two polymorphic forms of the oxathiin systemic fungicide active carboxine
Source: Acta Crystallogr E Crystallogr Commun. 2018 Nov 9;74(Pt 12):1741–5. doi: 10.1107/S2056989018015451 (PMC6281101; doi:10.1107/S2056989018015451)
Supplement: Supplementary file 1 [file e-74-01741-sup6.docx]

Table of solvents used in the truncated polymorph screen.

Solvent	Result
Hexane	Amorphous / Form 1
Toluene	Form 1
MTBE	Amorphous / Form 1
Ethyl Acetate	Form 1
IPA	Form 1
THF	Amorphous / Form 1
Acetone	Form 1
Methanol	Form 2
Acetonitrile	Form 1
Nitromethane	Form 1
Water	Form 1
5%EtOH/Water	Form 1
